# Supplementary material for: Causal interplay between lactose intolerance and gut microbiota: a combined bidirectional Mendelian randomization and in vivo validation study
Source: Front Nutr. 2026 Jun 1;13:1803337. doi: 10.3389/fnut.2026.1803337 (PMC13265576; doi:10.3389/fnut.2026.1803337)
Supplement: Supplementary file 1 [file Data_Sheet_1.zip › supplementary materials/Forward/leave one out cross validation/ebi-a-GCST90027514.finngen_R12_E4_LACTONAS.pdf]

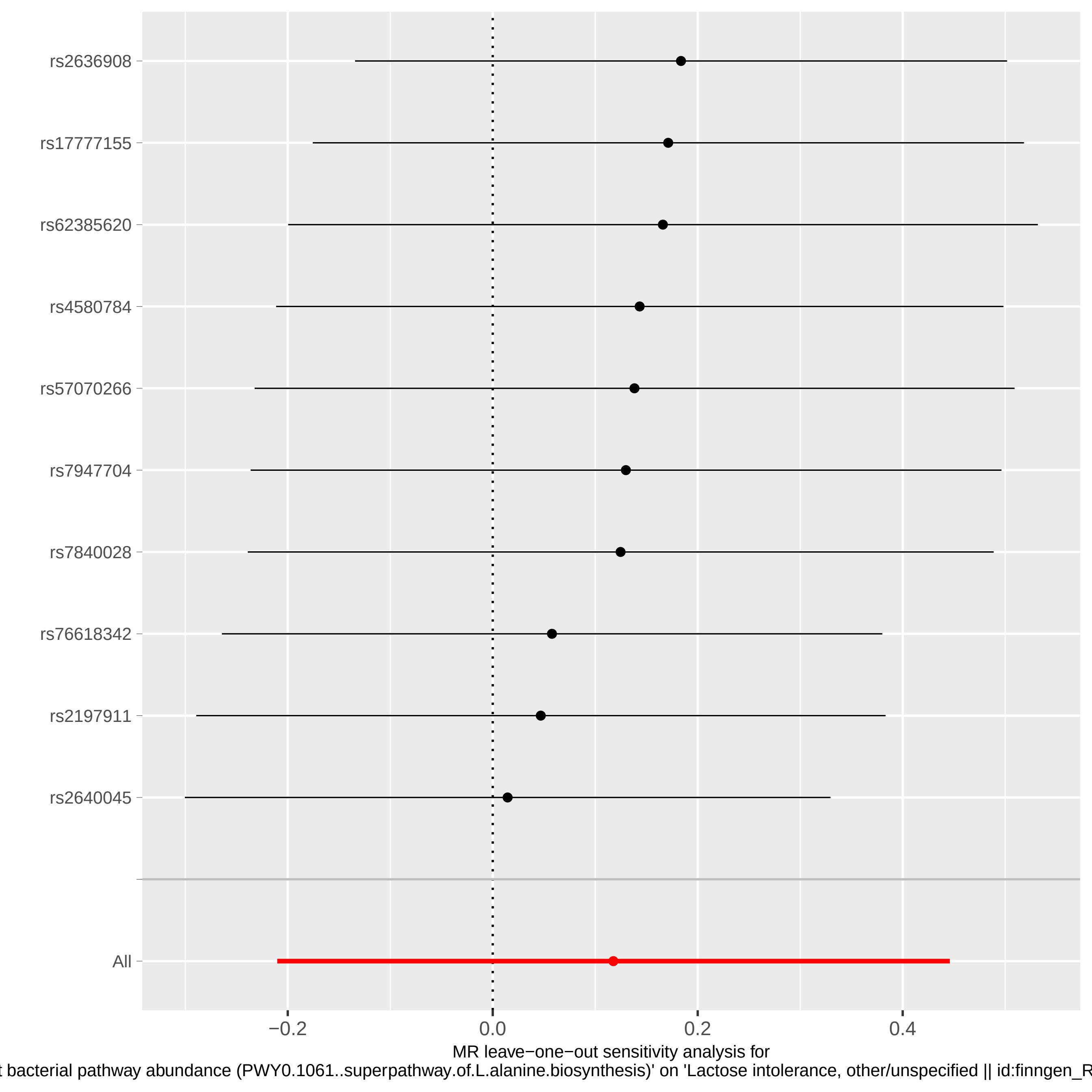
rs2636908
rs17777155
rs62385620
rs4580784
rs57070266
rs7947704
rs7840028
rs76618342
rs2197911
rs2640045
All
−0.2
0.0
0.2
0.4
MR leave−one−out sensitivity analysis for
t bacterial pathway abundance (PWY0.1061..superpathway.of.L.alanine.biosynthesis)' on 'Lactose intolerance, other/unspecified || id:finngen_R
